# The measurement of three dimensional dose distribution of a ruthenium‐106 ophthalmological applicator using magnetic resonance imaging of BANG polymer gels^1^

**DOI:** 10.1120/jacmp.v2i2.2617

**Published:** 2001-03-01

**Authors:** Maria F. Chan, Albert YC Fung, Yu‐Chi Hu, Chen‐Shou Chui, Howard Amols, Marco Zaider, David Abramson

**Affiliations:** ^1^ Department of Medical Physics Memorial Sloan‐Kettering Cancer Center 1275 York Ave New York New York 10021; ^2^ Department of Ophthalmology New York Presbyterian Hospital New York New York 10021

**Keywords:** ophthalmologic applicator, MRI, gel dosimetry, brachytherapy, ocular melanoma, retinoblastoma, eye cancer

## Abstract

The BANG (MGS Research Inc., Guilford, CT) polymer gel has been used as a dosimeter to determine the three‐dimensional (3D) dose distribution of a ruthenium‐106 (Ru‐106) ophthalmologic applicator. An eye phantom made of the BANG gel was irradiated with the Ru‐106 source for up to 1 h. The phantom and a set of calibration vials were scanned simultaneously in a GE 1.5 T MR imager using the Hahn spin‐echo pulse sequence with a TR of 2000 ms and two TEs of 20 ms and 100 ms. The $T_{2}$ values were evaluated on a pixel‐by‐pixel basis using custom‐built software on a DEC alpha workstation and converted to dose using calibration data. Depth doses and isodose lines of the Ru‐106 eye‐plaque were generated. It is concluded that the BANG gel dosimetry offers the potential for measuring the 3D dose distributions of an ophthalmologic applicator, with high spatial resolution and relatively good accuracy.

PACS number(s): 87.66.–a, 87.90.+y

## BACKGROUND

**Figure 1:**
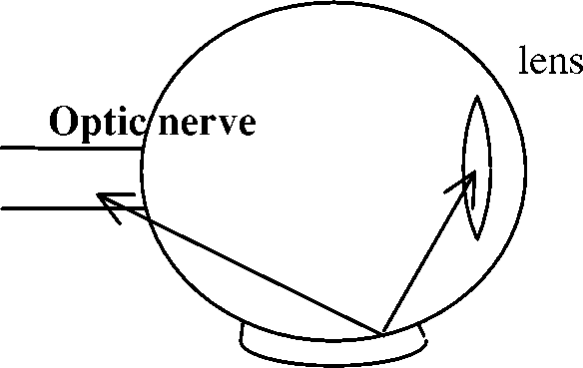
Gamma rays from an I‐125 eye plaque caused significant dose to the lens and optic nerve.

Brachytherapy has been used to treat ocular cancers for 70 years. The ongoing collaborative ocular melanoma study (COMS) randomized trial compares survival for enucleated vs. irradiated patients and has been accruing patients since 1986.^1^ That study and most of the eye plaques used worldwide utilize iodine‐125 (I–25). However, an increasing number of clinical centers have been using ruthenium‐106 (Ru‐106) eye plaques, especially for melanomas^2^ and retinoblastomas.^3^ Since I–125 is a gamma emitter, direct and scattered radiation from I–125 reaches the lens and the optic nerve (critical organs) and causes a significant dose (Fig. 1). A beta emitter with a finite range, such as Ru‐106, should minimize this problem.

**Figure 2:**
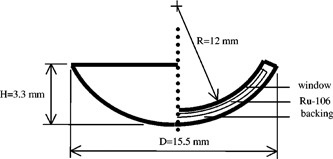
Structure of a typical Ru‐106 eye plaque.

Ru‐106 has a half‐life of 373.59 days and beta decays to Ru‐106 which emits a 3.54‐MeV beta (8‐mm range in tissue) upon decaying (30‐sec half‐life) to stable Pd‐106. The Ru‐106 half‐life permits clinical utility of the applicator for 1‐2 years. Figure 2 shows the structure of an eye‐plaque consisting of a thin film of Ru‐106 encapsulated in a sheet of pure silver, with a total thickness of 1 mm. The concave window is 0.1‐mm thick, and the rear surface absorbs 95% of the beta radiation. The plaque is manufactured by BEBIG,^4^ and various diameters and activities are available. The plaque that we have obtained is of type CCA, with a diameter of 15.5 mm and manufacturer's activity of 12.1 MBq±30% which gives a surface dose rate of about 12 cGy/min. An inactive plexiglass dummy of the same geometry is available for practice handling and localization during surgery. During measurement, wipe tests were performed between every procedure to ensure that radiation leakage did not happen.

A system that employs a chemical dosimeter (ferrous sulfate solution) fixed in an aqueous gel and evaluated with magnetic resonance imaging (MRI) has been proposed to verify the 3D radiation dose mapping.^5^ ^–^ ^9^ More recently, the BANG polymer gel in combination with an MRI has been shown to record and accurately map 3D dose distributions with high spatial resolution and precision.^10^ ^–^ ^14^ In this study, the BANG gel has been used as a 3D dosimeter for a Ru‐106 ophthalmologic applicator. Since the proton relaxation rate $\left(R_{2}\right)$ of the gel changes as a function of the absorbed dose, MR scans of the irradiated gel can be used to generate 3D dose distributions.

## METHODS AND MATERIALS

The BANG gel was used to make a flat gel phantom impressed on one side with two dummy eye plaques. The gel phantom was covered by paraffin to minimize oxygen and kept in a dark environment before irradiation. There were also five glass vials (22‐mm diameter) containing the gel for calibration. The gel was kept at room temperature for 8 h before irradiation. The gel phantom was irradiated with the Ru‐106 source in two separate sessions in a glove bag filled with pure nitrogen, one for 1 h and the other for 0.3 h, in order to evaluate the dose distribution at different regions. At the same time, the vials were irradiated with 6‐MV photon beams using a Varian Clinac 600C linear accelerator. All vials were wrapped with bolus material during irradiation and positioned at 100‐cm source‐to‐surface distance (SSD). Calibration was performed by irradiating the five vials to four different dose levels (control, 100, 200, and 300 cGy). All MR images were acquired with a 1.5‐T MR scanner equipped with an extremity coil after 15 days of irradiation. Two single echo pulse sequences (TR=2000 ms, TE=20 ms and 100 ms) were acquired. The phantom and calibration vials were scanned simultaneously. Imaging parameters included an 8‐cm field‐of‐view, a 256×256 matrix with a slice thickness of 3 mm, and the number of excitations for the two. The total imaging time was 1.5 h in this experiment. The images obtained with the MR scanner were transferred to a DEC alpha workstation.

## RESULTS

**Figure 3:**
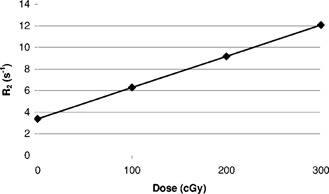
The linearity $\left(r^{2}=0.997\right)$ of the gel irradiated by 6‐MV *x* rays and $R_{2}$ measured using custom‐built software on a DEC alpha workstation.

The proton relaxation rate, $R_{2}$, was calculated with the use of a two‐parameter fit of a signal equation $R_{2}=[{\left({\text{TE}}_{2}-{\text{TE}}_{1}\right)}^{-1}]*\text{log}[S({\text{TE}}_{1})*S{\left({\text{TE}}_{2}\right)}^{-1}]$, and a custom‐built software on the DEC alpha workstation. The values of $R_{2}$ measured for each dose level were averaged among the pixel values within the region of interest (ROI). The results are shown in Fig. 3. The plot (Fig. 3) of the $R_{2}$ vs. dose in the calibration vials gave a straight line $\left(r^{2}=0.997\right)$ with a slope of $0.02899s^{-1}{\text{cGy}}^{-1}$ with an intercept of $3.383s^{-1}$. The standard deviation was of the order of less than 5%, except in the highest dose region where it was near 25%. The curve is a linear fit of $R_{2}$ and the dose from the calibration data. A calibration curve should be generated for each measurement because of variations in temperature, the composition of the gel, and the imaging process.

**Figure 4:**
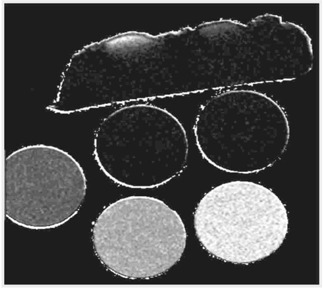
Spatial radiation dose reconstruction from MR signals with custom‐built software on a DEC alpha workstation. The five vials were irradiated to four dose levels (0, 100, 200, and 300 cGy).

**Figure 5:**
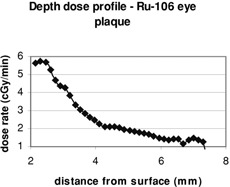
Depth doses of Ru‐106 eye plaque (dose rate vs. distance from surface) generated by the BANG gel.

**Figure 6:**
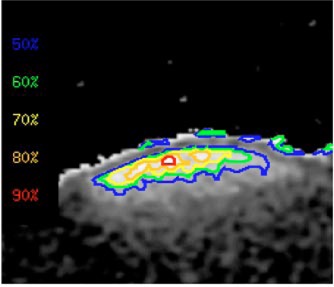
(Color) To reduce the noises and artifacts in the dose array, the dose threshold was set to 300. If the pixel value is 300, it means 300 cGy. The gel was irradiated for 60 min, hence the dose rate should be 5 cGy/min; 90% is 4.5 cGy/min; 80% is 4 cGy/min, respectively.

The signals at each pixel position from the images at two different TE values were converted to $R_{2}$ at that pixel through a custom‐built computer program on the alpha workstation. Then the $R_{2}$ value was converted to dose using the calibration curve, resulting a dose image (Fig. 4). Such images give both direct visual information of the dose distribution and the absolute dose values. The spatial resolution is 0.3 mm for each pixel. Figure 4 also shows MR artifacts created at the interfaces of different materials (gel and air) due to the differences in chemical shifts and magnetic susceptibilities. Therefore, the use of gel dosimetry for surface regions is not recommended. After completion of the dose conversion process, depth doses and isodose lines of the Ru‐106 eye plaque were generated. The depth doses in the region of 2 to 7.5 mm from the surface of the eye phantom are depicted in Fig. 5. Figure 6 shows the isodose lines (90% represents dose rate of 4.5 cGy/min, i.e. pixel value of 270 cGy for 60 min of irradiation). To reduce the noises and artifacts in the dose array, the dose threshold was set to 300 since any pixel with value larger than 300 cGy could be considered as noise.

## CONCLUSIONS

Although the BANG polymer gel dosimetry is somewhat expensive, it provides the ability to map the 3D dose distribution with high spatial resolution. Besides depending on the actual performance of the MR imager, the overall uncertainty in the calculated $R_{2}$ values is highly affected by the environmental temperature, light, and oxygen during the experiment. It is necessary to keep the phantom and vials at the same environment throughout the experiment in order to determine the dose quantitatively. During irradiation of the calibration vials, the use of bolus material instead of water as medium provides an excellent temperature control, which reduces the differences between the measured dose and the predicted dose in the phantom.
